# Effect of concomitant atrial septal defect on left ventricular function in adult patients with unrepaired Ebstein’s anomaly: a cardiovascular magnetic resonance imaging study

**DOI:** 10.1186/s12968-023-00976-3

**Published:** 2023-12-07

**Authors:** Xi Liu, Yue Gao, Zhen Wang, Rui Shi, Wen-Lei Qian, Meng-Ting Shen, Ying-Shi Sun, Zhi-Gang Yang

**Affiliations:** 1 Department of Radiology, West China Hospital, Sichuan University, 37# Guo Xue Xiang, Chengdu, 610041 Sichuan China; 2 Key Laboratory of Carcinogenesis and Translational Research (Ministry of Education/Beijing), Department of Radiology, Peking University Cancer Hospital and Institute, 52# Fu Cheng Road, Hai Dian District, Beijing, 100142 China

**Keywords:** Ebstein’s anomaly, Cardiovascular magnetic resonance, Atrial septal defect, Myocardial strain

## Abstract

**Background:**

Due to the heterogeneity of anatomic anomalies in Ebstein’s anomaly (EA), particularly in the subset of patients with atrial septal defect (ASD), hemodynamic changes, which ultimately cause left ventricular (LV) deterioration remain unclear. The current study aimed to investigate the effect of concomitant ASD on LV function using cardiovascular magnetic resonance (CMR) imaging in patients with EA.

**Methods:**

This study included 31 EA patients with ASD, 76 EA patients without ASD, 35 patients with simple ASD and 40 healthy controls. Left/right ventricular (RV, the RV was defined as a summation of the functional RV and atrialized RV in EA patients) volumes and functional parameters, LV strain parameters, and echocardiogram indices were compared among the four groups. Associations between variables were evaluated via Spearman or Pearson correlation analyses. The association between risk factors and LV ejection fraction (EF) was determined via multivariate linear regression analysis.

**Results:**

Both EA patients and ASD patients had a higher RV/LV end-diastolic volume (RVEDV/LVEDV) as well as lower LV and RV ejection fractions (LVEF/RVEF) compared to healthy controls (all p < 0.05). Moreover, the EA patients with ASD had a significantly higher RVEDV/LVEDV and a lower LVEF and RVEF than those without ASD (all p < 0.05). Multivariate linear regression analysis revealed that the presence of ASD was independently associated with LVEF (β = − 0.337, p < 0.001). The RVEDV/LVEDV index was associated with LVEF (r = − 0.361, p < 0.001). Furthermore, the LV longitudinal peak diastolic strain rate (PDSR) was lower in EA patients with ASD than those without ASD, patients with simple ASD, and healthy controls (p < 0.05).

**Conclusion:**

Concomitant ASD is an important risk factor of LV dysfunction in patients with EA, and diastolic dysfunction is likely the predominate mechanism related to LV dysfunction.

**Supplementary Information:**

The online version contains supplementary material available at 10.1186/s12968-023-00976-3.

## Background

Ebstein’s anomaly (EA) is a rare congenital malformation caused by tricuspid valve (TV) delamination failure. The dysplastic and tethered TV (the septal and/or posterior leaflet) is offset toward the apex of the right ventricle (RV), with subsequent atrialization of the RV inlet. Any remaining inlet portion below the tethered TV, the trabeculated RV, and the RV outflow tract comprise the functional right ventricle (fRV) [1]. These anatomic abnormalities often lead to progressive tricuspid regurgitation (TR) and right ventricular volume overload (RVVO), which in turn induce progressive dilation and dysfunction of the RV [2, 3]. In addition, an interatrial communication such as an atrial septal defect (ASD) is common, and the left-to-right shunt at the atrial level may further aggravate RVVO [4]. Due to increased right heart pressures and declining compliance, right-to-left shunting via the ASD can occur and lead to progressive cyanosis [5]. Although EA is primarily an RV disease, the shape and function of the left ventricle (LV) is also affected [6]. However, risk factors associated with LV dysfunction in EA remain elusive, in part due to the heterogeneity of the disease, such as the presence or absence of an ASD.

Cardiovascular magnetic resonance (CMR) imaging is a complementary imaging modality to echocardiography. Furthermore, it has emerged as the gold standard for evaluating the function and size of the LV and RV, which can help in risk stratification and treatment decision-making in patients with EA [7, 8]. Therefore, the current study aimed to use CMR combined with echocardiographic indices to investigate the effect of concomitant ASD on LV function in patients with EA.

## Methods

### Study population

Patients diagnosed with EA based on echocardiogram results in our registry database from July 2013 to November 2022 were evaluated [9]. This study retrospectively enrolled patients who underwent preoperative CMR imaging. The exclusion criteria were as follows: patients aged under 18 years, those with a history of cardiac surgery, those with other complex congenital cardiac anomalies (such as congenitally corrected transposition of the great arteries), those with left ventricular non-compaction cardiomyopathy, and those with inadequate data on CMR imaging due to poor image quality. For comparison, we also included a control group of 40 healthy subjects who underwent CMR examination due to clinical suspicion of cardiovascular disease. Control subjects were excluded if they had clinical evidence of cardiovascular disease or arrhythmia or inadequate data on CMR imaging due to poor image quality. In addition, we retrospectively included 35 patients with ASD (diagnosed via echocardiography) who underwent preoperative CMR imaging. The exclusion criteria were as follows: patients aged under 18 years, those with other congenital cardiac anomalies, and those with inadequate CMR studies with poor image quality.

The current study was approved by the Biomedical Research Ethics Committee of our hospital. The electronic medical records of the patients were reviewed, and data on the New York Heart Association (NYHA) functional classification and echocardiographic parameters (such as presence of ASD, TR grade, and length of TV leaflet offset) were recorded.

### MR protocol and image analysis

CMR imaging was performed using a 3 T MR scanner (Tim Trio and Skyra, Siemens Healthineers, Erlangen, Germany). All participants were examined according to the standardized imaging protocol for congenital heart disease [10]. Cine images were acquired with a balanced steady-state free-precession sequence (TR: 2.9/3.4 ms; TE: 1.25 /1.3 ms; flip angle: 50/40°; slice thickness: 8 mm; field of view: 319 × 249/339 × 284 mm^2^; matrix size 256 × 166/256 × 144). Late gadolinium enhancement (LGE) imaging was conducted for 10–15 min after the intravenous administration of gadolinium contrast (0.15 mmol/kg) using a T1-weighted inversion recovery turboFLASH sequence (TR: 650/500 ms; TE: 1.43/1.24 ms, flip angle: 50/40°, slice thickness: 8 mm, field of view: 340 × 255/340 × 233 mm^2^, matrix size: 256 × 192/ 256 × 125).

All CMR imaging data were analyzed using a commercially available software (cvi42 version 5.11.2, Circle Cardiovascular Imaging Inc., Calgary, Canada). The LV volume and functional parameters including end-diastolic volume, end-systolic volume, stroke volume, and ejection fraction (EF) were acquired, as described in a previous study [11]. The RV (functional RV + atrialized portion of RV in EA) volume and functional parameters were acquired by delineating corresponding endocardial contours manually in serial short-axis slices at the end-diastolic and end-systolic phases, as described in previous studies [12, 13]. To analyze LV myocardial strain, short-, long-axis two-chamber, and four-chamber slices were loaded into the tissue tracking module. LV endocardial and epicardial contours were delineated, as described in our previous study [11]. Subsequently, the software automatically tracked the contour throughout the whole cardiac cycle. In addition, the propagated borders were checked and manually adjusted if it was considered. The LV global myocardial strain parameters including radial, circumferential, and longitudinal peak strain (PS), peak systolic strain rate (PSSR), and peak diastolic strain rate (PDSR) were then estimated automatically. LGE was visually identified as positive (LGE +) if there were hyperintense regions within the myocardium at the short- and long-axis views.

### Statistical analysis

All statistical analyses were performed using SPSS statistical software (version 23.0; SPSS Inc., Chicago, Illinois, USA) and GraphPad Prism (version 8.0.1, GraphPad Software Inc., San Diego, California, USA). Continuous variables were presented as means ± standard deviations or as medians and interquartile ranges. Normality was evaluated using the Kolmogorov–Smirnov test, and the homogeneity of variance was assessed using the Levene’s test. One-way analysis of variance was used to compare variables among the healthy control, ASD, EA with ASD, and EA without ASD groups. Then, post hoc analysis was performed using the Bonferroni’s correction. Binary variables were analyzed using the cross tabs chi-square test. Associations between variables were investigated via Spearman or Pearson correlation analyses. Further, univariate factors related to the LVEF with a p-value of < 0.1 were then included in the stepwise multivariate analysis. A p-value of < 0.05 was considered statistically significant.

## Results

### Baseline characteristics

**Fig. 1:**
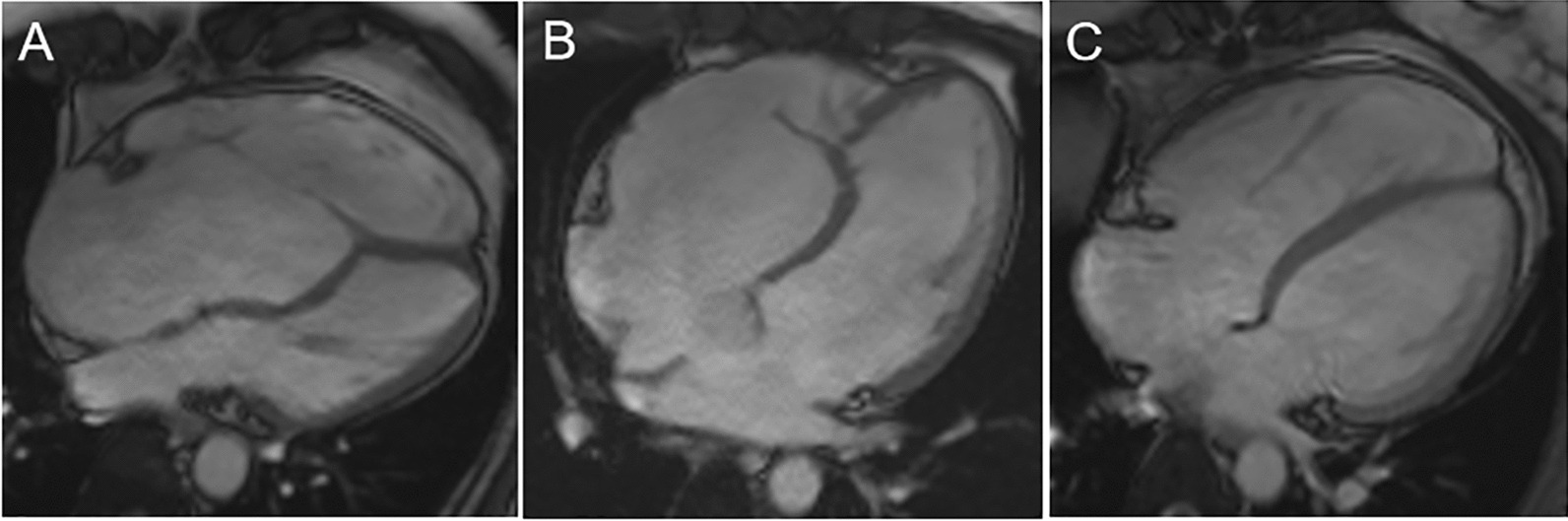
Representative four-chamber cardiac magnetic resonance images at the end-diastolic phases in EA patients without ASD (**A**), EA patients with ASD (**B**), and ASD patients (**C**)

The final study cohort comprised 107 patients with EA, 35 patients with simple ASD, and 40 healthy controls. Atrial septal defects were confirmed on echocardiogram in patients with EA [14]. Among 107 patients with EA, 31 (29%) had concomitant ASD (Fig. 1). Patients with EA were divided into the EA with ASD group (n = 31) and the EA without ASD group (n = 76). The baseline characteristics of the EA patients, ASD patients and normal controls are presented in Table 1.

**Table 1:** Baseline Characteristics of Control Individuals, ASD Patients, EA Patients without or with ASD

	Normal n = 40	ASD n = 35	EA without ASD n = 76	EA with ASD n = 31	*p* value
Age, y	45.4 ± 9.7	43.5 ± 13.7	38.5 ± 13.3*	41.0 ± 12.9	0.006
Male Gender, n (%)	14 (35%)	10 (29%)	27 (36%)	9 (29%)	0.740
BSA, m^2^	1.57 ± 0.11	1.54 ± 0.13	1.54 ± 0.12	1.52 ± 0.11	0.152
BMI, kg/m^2^	21.9 ± 1.7	21.9 ± 3.5	21.4 ± 2.5	21.4 ± 1.9	0.557
Heart rate, bpm	76 ± 7.9	80 ± 16	80 ± 14	81 ± 9.7	0.246
Cyanosis, n (%)	–	2 (5.7%)	5 (6.6%)	13 (42%)^†‡^	< 0.001
LGE +, n (%)	–	14 (40%)	14 (18%)	16 (52%)^‡^	0.003
Pericardial effusion, n (%)	–	22 (63%)	42 (55%)	17 (55%)	0.730
NYHA functional class, n (%)					
I	–	4 (11%)	12 (16%)	3 (9.7%)	0.347
II	–	23 (66%)	47 (62%)	19 (61%)	
III	–	7 (20%)	16 (21%)	6 (19%)	
IV	–	1 (2.9%)	1 (1.3%)	3 (9.7%)	
Echocardiogram measures					
Posterior leaflet offset (mm/m^2^)	–	–	26 ± 7.3	31 ± 12	< 0.001
Septal leaflet offset (mm/m^2^)	–	–	14 (11–18)	20 (16–24)	< 0.001
Tricuspid regurgitation, n (%)					
Mild/moderate	–	23 (66%)	35 (46%)^†^	6 (19%)^†‡^	< 0.001
Severe	–	7 (20%)	41 (54%)^†^	25 (81%)^†‡^	

Data given as the mean ± SD or median (25th, 75th percentile), *p* value for trend between groups. Pairwise comparisons using Bonferroni correction: ^*^p < 0.05 vs controls; ^†^*p* < 0.05 vs ASD; ^‡^*p* < 0.05 vs EA without ASD. EA, Ebstein’s anomaly; ASD, atrial septal defect; BSA, body surface area; BMI, body mass index; LGE, lategadolinium enhanced; NYHA, New York Heart Association

As shown in Table 1, most of the EA patients and ASD patients were classified as NYHA functional classes II or III, however, no significant differences were observed between the EA subgroups. In addition, we reviewed the TR grade and the length of TV leaflet offset of all patients with EA based on the echocardiographic results. The two EA subgroups had more severe TR than the ASD group (all p < 0.05). The number of patients with severe TR in the EA with ASD group was significantly higher than that of patients with severe TR in the EA without ASD group (81% vs 54%). Moreover, the posterior and septal valve leaflets of the malformed and downwardly displaced TV into the RV were more severe in the EA with ASD group than in the EA without ASD group (31 ± 12 vs. 26 ± 7.3, 20 (16–24) vs. 14 (11–18), respectively; all p < 0.05).

### CMR imaging results

**Fig. 2:**
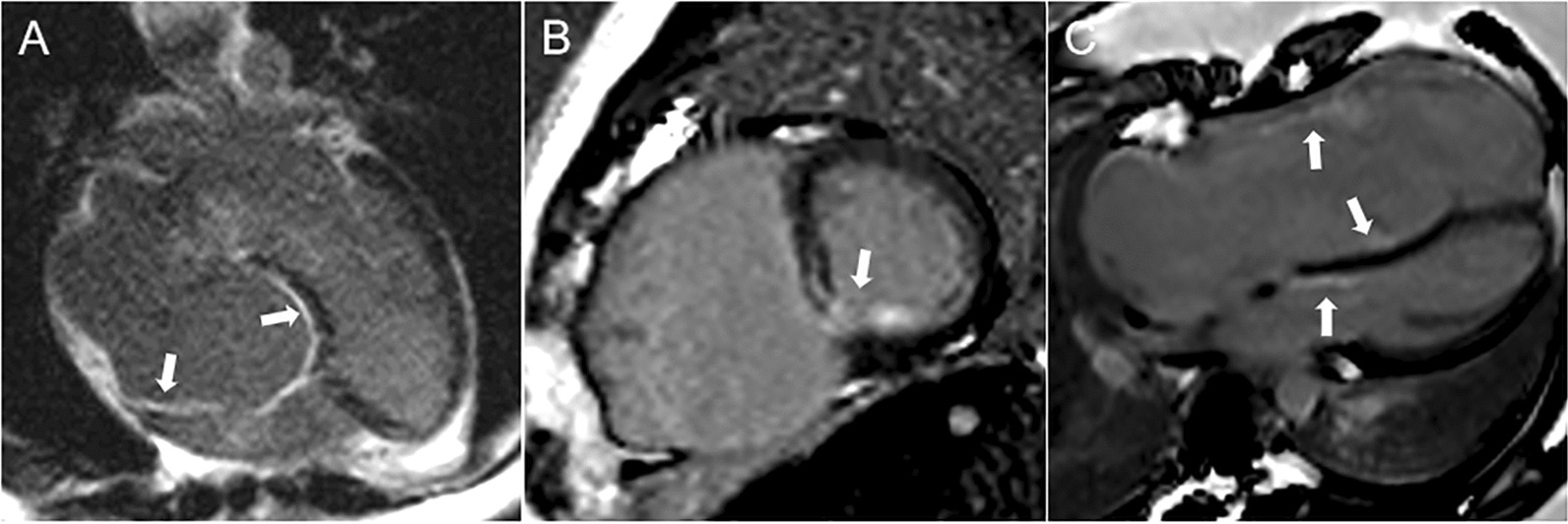
Representative images of LGE in patients with EA: Endocardial LGE in atrialized right ventricle (**A**). Endocardial LGE in the middle septum (**B**). LGE in both the basal septum and aRV endothelium (**C**)

On CMR images, LGE was identified in 30/107 (28%) of EA patients, mainly occurred at subendocardium of the RV and/or basal and middle septum (Fig. 2). The EA with ASD group had a higher incidence of LGE than the EA without ASD group (52% vs. 18%, p < 0.05).

**Fig. 3:**
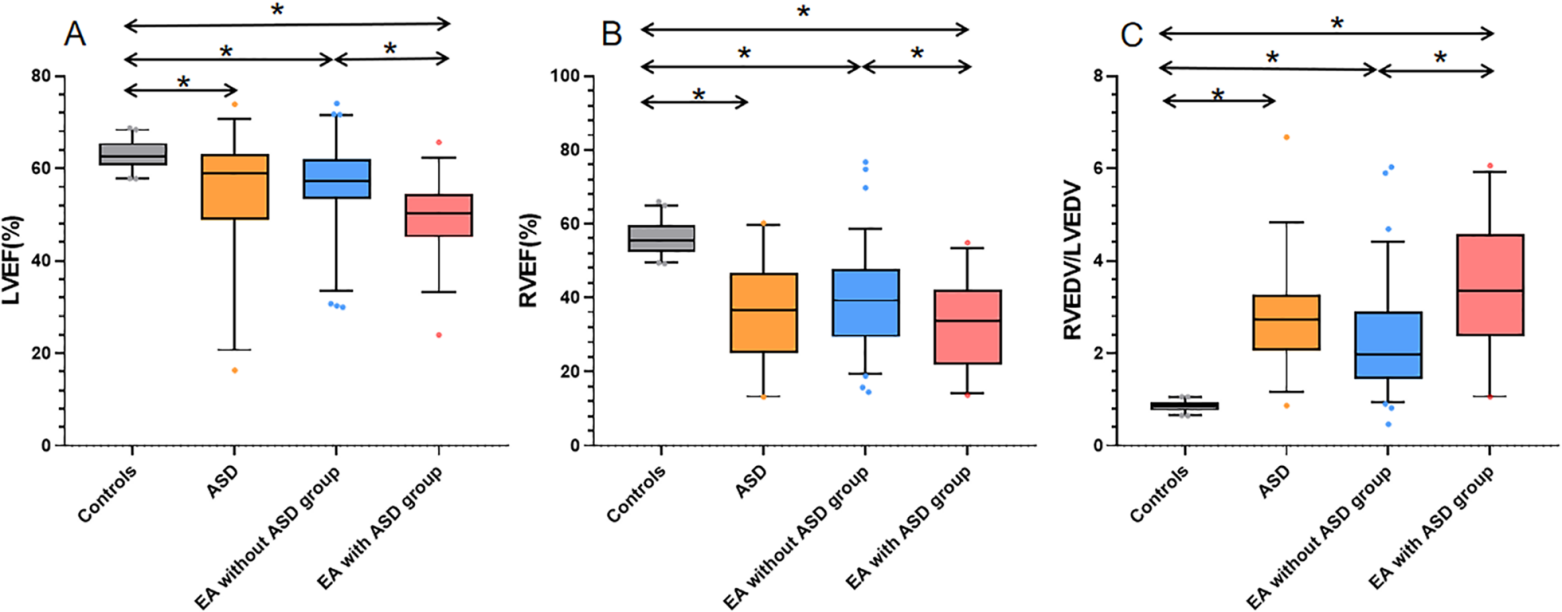
Differences in LVEF (**A**), RVEF (**B**), and RVEDV/LVEDV (**C**) among healthy controls, ASD patients, EA patiens without ASD, and EA patients with ASD. The dots indicate values outside the interquartile range, *p < 0.05

Table 2 depicts data on the volume and functional parameters of LV/RV. There was no significant difference in terms of the LVEDV index (LVEDVI, indexed to BSA) between healthy controls, EA patients without ASD, and simple ASD patients. However, EA patients with ASD had a significantly lower LVEDVI than healthy controls and EA patients without ASD (p < 0.05). Patients with EA and those with ASD had a higher RVEDVI and RVEDV/LVEDV than healthy controls (all p < 0.05). In addition, the EA with ASD group had a significantly higher RVEDVI and RVEDV/LVEDV than the EA without ASD group (all p < 0.05). Patients with EA and those with ASD had a lower LVEF and RVEF than healthy controls (all p < 0.05). In addition, the EA with ASD group had a significantly lower LVEF and RVEF than the EA without ASD group (all p < 0.05) (Fig. 3).

**Table 2:** CMR Parameters between Normal Individuals, ASD Patients, EA Patients without or with ASD

	Normal n = 40	ASD n = 35	EA without ASD n = 76	EA with ASD n = 31	*p* value
LVEDVI, ml/m^2^	76 (72–81)	70 (64–76)	75 (67–86)	63 (54–80)^*‡^	0.006
LVESVI, ml/m^2^	28 (26–30)	29 (23–37)	33 (26–39)^*^	31 (27–38)	0.078
LVSVI, ml/m^2^	49 ± 5.5	39 ± 12^*^	44 ± 12	34 ± 9.4^*‡^	< 0.001
LVEF, %	64 (61–65)	59(49–63)^*^	57 (53–62)^*^	50 (45–55)^*‡^	< 0.001
RVEDVI, ml/m^2^	62 (58–69)	185 (143–219)^*^	155 (114–199)^*^	205 (168–277)^*‡^	< 0.001
RVESVI, ml/m^2^	28 (24–31)	108 (86–144)^*^	95 (64–137)^*^	129 (91–193)^*‡^	< 0.001
RVSVI, ml/m^2^	35 (31–40)	67 (45–87)^*^	58 (42–73)^*^	60 (41–89)^*^	0.666
RVEF, %	55 (52–60)	37 (25–47)^*^	39 (29–48)^*^	34 (22–42)^*‡^	< 0.001
RVEDV/LVEDV	0.84 (0.8–0.9)	2.7 (2.1–3.3)^*^	2.0 (1.4–2.9)^*^	3.4 (2.4–4.6)^*‡^	< 0.001

Data given as the mean ± SD or median (25th, 75th percentile), *p* value for trend between groups. Pairwise comparisons using Bonferroni correction: *p < 0.05 vs controls; ^‡^*p* < 0.05 vs EA without ASD;. LV, left ventricular; RV, right ventricular; EDV, end diastolic volume; ESV, end systolic volume; SV, stroke volume; EF, ejection fraction; I, indexed to BSA

**Fig. 4:**
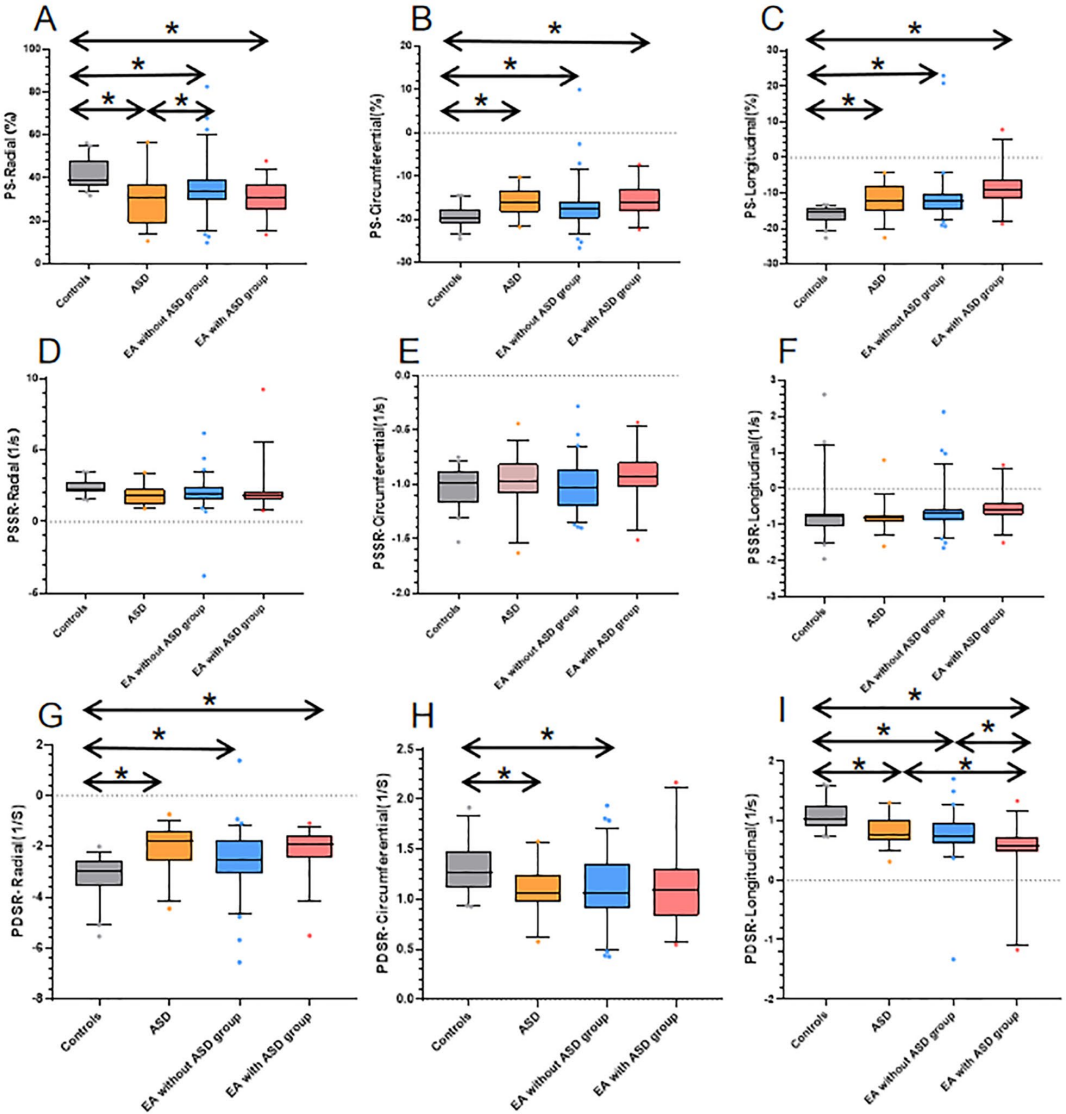
Differences in global radial, circumferential, and longitudinal PS (**A**–**C**), PSSR (**D**–**F**), and PDSR (**G**–**H**) among healthy controls, ASD patients, EA patients without ASD, and EA patients with ASD. The dots indicate values outside the interquartile range, *p < 0.05

Table 3 presents the LV global strain parameters of all participants. Compared with the normal controls, the radial, circumferential and longitudinal PS of patients with EA and those with ASD significantly reduced (all p < 0.05). In addition, the radial, circumferential, and longitudinal PDSR of patients with EA and those with ASD, except for the circumferential PDSR of the EA with ASD group, decreased (all p < 0.05). Moreover, the EA with ASD group had a significantly lower longitudinal PDSR than the non-ASD and simple ASD groups (all p < 0.05). However, there was no significant difference in terms of PSSR parameters among the four groups (Fig. 4).

**Table 3:** Left Ventricle Strain Parameters Difference between Normal Individuals, ASD Patients, EA Patients without or with ASD

	Normal n = 40	ASD n = 35	EA without ASD n = 76	EA with ASD n = 31	*p* value
PS (%)					
Radial	39 (36 to 48)	30 (19 to 37)^*^	34 (30 to 39)^*†^	30 (25 to 37)^*^	< 0.001
Circumferential	− 20 (− 21 to − 18)	− 16 (− 18 to − 13)^*^	− 18 (− 20 to − 16)^*^	− 16 (− 18 to − 13)^*^	0.001
Longitudinal	− 15 (− 18 to − 14)	− 12 (− 15 to − 7.8)^*^	− 12 (− 15 to − 10)^*^	− 9.3 (− 12 to − 6.3)^*^	< 0.001
PSSR (1/s)					
Radial	2.3 (2.1 to 2.7)	1.8 (1.2 to 2.3)	2.0 (1.6 to 2.4)	1.8 (1.6 to 2.1)	0.096
Circumferential	− 1.0 (− 1.2 to − 0.9)	− 1.0 (− 1.1 to − 0.8)	− 1.0 (− 1.2 to − 0.9)	− 0.9 (− 1.0 to − 0.8)	0.169
Longitudinal	− 0.8 (− 1.0 to − 0.7)	− 0.8 (− 0.9 to − 0.7)	− 0.7 (− 0.9 to − 0.6)	− 0.6 (− 0.7 to − 0.4)	0.159
PDSR (1/s)					
Radial	− 3.0 (− 3.6 to − 2.6)	− 1.8 (− 2.6 to − 1.4)*	− 2.6 (− 3.1 to − 1.7)^*^	− 1.9 (− 2.4 to − 1.6)^*^	< 0.001
Circumferential	1.3 (1.1 to 1.5)	1.1 (1.0 to 1.3)*	1.1 (0.9 to 1.4)*	1.1 (0.8 to 1.3)	0.004
Longitudinal	1.0 (0.9 to 1.3)	0.8 (0.7 to 1.0)*	0.8 (0.6 to 1.0)*	0.6 (0.5 to 0.7)*^†‡^	< 0.001

Data given as the median (25th, 75th percentile), *P* value for trend between groups. Pairwise comparisons using Bonferroni correction: *p < 0.05 vs controls; ^†^*p* < 0.05 vs ASD; ^‡^*p* < 0.05 vs EA without ASD. PS, peak strain; PSSR, peak systolic strain rate; PDSR, peak diastolic strain rate

### Correlation between clinical characteristics and the RVEDV/LVEDV index, LVEF, and RVEF

**Fig. 5:**
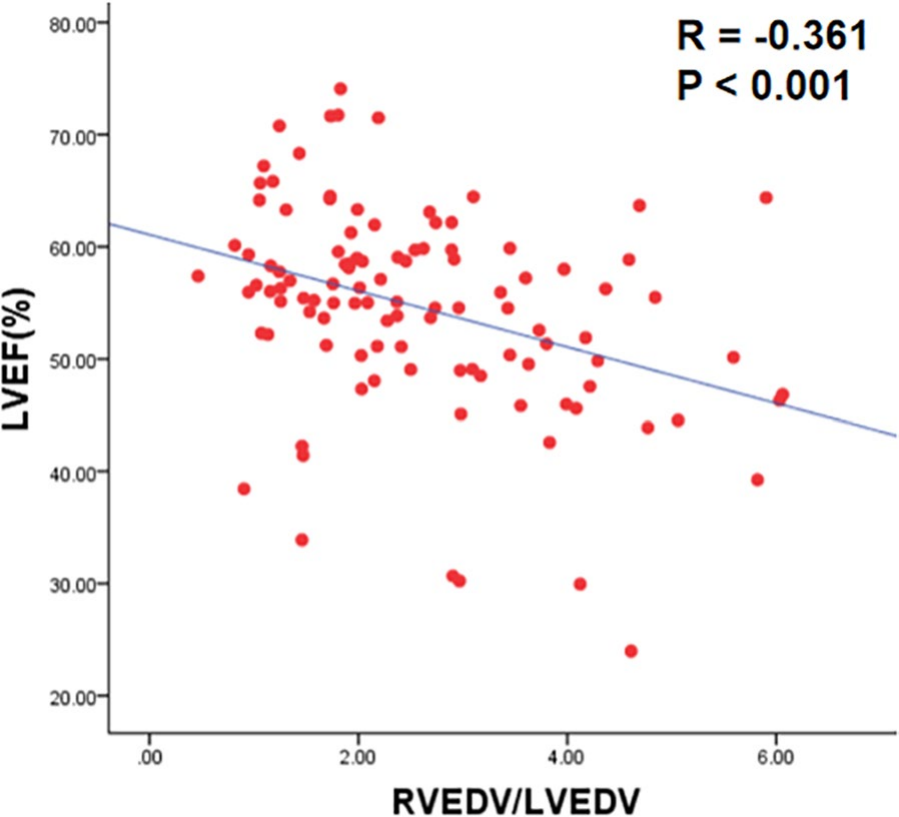
Association between RVEDV/LVEDV and LVEF

As shown in Table 4, there were weak to moderate correlations between the presence of ASD, posterior leaflet offset, TR grade, and RVEDV/LVEDV index in patients with EA (r = 0.378, p < 0.001; r = 0.439, p < 0.001; r = 0.582, p < 0.001; respectively). The length of posterior leaflet offset was associated with RVEF (r = − 0.330, p = 0.001), and the TR grade was associated with LVEF (r = − 0.323, p = 0.001). Besides, we have found that RVEDV/LVEDV index was associated with LVEF (r = − 0.361, p < 0.001) (Fig. 5). As shown in Table 5, multivariate linear regression analysis revealed that the presence of ASD was independently associated with LVEF (β = − 0.337, p < 0.001).

**Table 4:** Correlation Analysis in EA Patients

	RVEDV/LVEDV		RVEF		LVEF	
	r	*p* value	r	*p* value	r	*p* value
Age	− 0.016	0.866	0.148	0.129	0.127	0.191
Cyanosis	0.211	0.029	− 0.260	0.007	− 0.123	0.207
ASD +	0.378	< 0.001	− 0.239	0.013	− 0.406	< 0.001
LGE +	0.135	0.166	− 0.246	0.011	− 0.244	0.011
Pericardial effusion	0.155	0.110	− 0.105	0.281	− 0.194	0.045
NYHA functional class	− 0.026	0.789	− 0.058	0.552	− 0.120	0.217
Tricuspid regurgitation	0.582	< 0.001	− 0.283	0.003	− 0.323	0.001
Septal leaflet offset	0.268	0.005	− 0.269	0.005	− 0.279	0.004
Posterior leaflet offset	0.439	< 0.001	− 0.330	0.001	− 0.237	0.014

**Table 5:** Multivariate linear regression of factors associated with LVEF

	Univariable		Multivariable	
	r	*p* value	β	*p* value
Age	0.127	0.191		
Gender	− 0.022	0.824		
ASD +	− 0.406	< 0.001	− 0.337	< 0.001
LGE +	− 0.244	0.011	− 0.140	0.155
Pericardial effusion +	− 0.194	0.045	− 0.213	0.020
Septal leaflet offset	− 0.279	0.004	− 0.157	0.110
Posterior leaflet offset	− 0.237	0.014	− 0.148	0.131
Tricuspid regurgitation	− 0.323	0.001	− 0.150	0.136

Factors with p < 0.1 in the univariable analysis were included in the multivariable analysis

## Discussion

The presentation of adult patients with EA varies from asymptomatic to severely symptomatic, and asymptomatic patients should be under close follow-up. The indication and appropriate timing of surgical intervention in EA remains a matter of debate [15]. Generally, surgical intervention is recommended for patients with evidence of progressive right heart enlargement and heart failure symptoms [16]. In patients with EA who presented with ASD, ASD closure in combination with surgical TV intervention or as an isolated transcatheter procedure could be performed. Although EA is primarily an RV disease, the shape and function of the LV are also affected [6]. If the LV function is severely depressed, traditional surgical correction is not possible, and heart transplantation should be considered. A recent study revealed that TV reconstruction must be performed before the deterioration of RVVO and LV function [17]. Therefore, earlier intervention is recommended to prevent LV dysfunction progression [18].

Left ventricular dysfunction, paradoxical septal movement, LV dyssynchrony, and LV myocardial fibrosis have been reported in patients with EA [13, 19, 20]. However, the mechanisms underlying the deterioration of LV function and their role in the development of heart failure are not completely understood. In addition, EA is frequently accompanied by ASD, thereby making hemodynamic changes leading to LV worsening even more elusive. Understanding the effect of concomitant ASD on LV function in patients with EA is helpful for clinical management and surgical treatment decision-making. Currently, CMR can provide surgeons with a more comprehensive assessment of morphological and functional information prior to surgery in EA. Therefore, the current study used CMR imaging combined with echocardiographic indices to investigate the effect of concomitant ASD on LV function in patients with EA.

Generally, echocardiography is the first line for TV imaging and that the TV is a difficult structure to adequately visualize, that frequently requires multimodality imagings (such as echocardiography and CMR). However, CMR imaging is more effective in quantifying the size and function of the right ventricle [7, 8]. It is challenging to define fRV on CMR images in terms of reproducibility because TV tracing can be difficult on cine images. This study recommended a simplified CMR-based index defined by the ratio of the total RV/LV volume, disregarding the ill-defined malformed TV borders. In the present study, we identified correlations of the RVEDV/LVEDV index with several indicators of EA severity, including TR grade, and the length of posterior leaflet offset. The total RVEDV/LVEDV ratio considers the transversal interaction of RV and LV, which may be a reliable and easy approach for evaluating EA severity.

Our study revealed that EA patients with ASD had a significantly higher RVEDV/LVEDV index than those without ASD, indicating that those patients with ASD had a more severe LV compression caused by an enlarged RV. Further, the RVEDV/LVEDV index was associated with LVEF. However, EA patients with ASD had a significantly higher rate of severe TR, which also increased the volume load of the RV. In addition, EA patients with ASD had a more severe tricuspid valve leaflet displacement and a higher incidence of LGE +, which indicated a more severe disease. Thus, it was challenging to conclude that differences in RV/LV size and function between the two EA subgroups were attributed to the presence of ASD. These variations might be caused by the fact that EA patients with ASD also had a more severe anatomic variant with more severe TR. To correct confounding factors affecting the LVEF, we conducted a multivariate linear regression analysis. The results showed that the presence of ASD was independently associated with LVEF, confirming that the presence of ASD is an extremely important risk factor of LV dysfunction.

TR and left-to-right shunt caused by ASD can lead to RVVO. In this study, the RVEDVI were significantly greater in EA patients and in ASD patients, which was not surprising. In some patients with EA, ASD was found, and could cause a right-to-left shunt owing to increased right heart pressures and compliance deterioration, which can contribute to right-side overload discharge [5]. However, our results showed that EA patients with ASD had a significantly greater RVEDVI than those without ASD. Therefore, ASD is an extremely important risk factor of RVVO in patients with EA. We speculated that prolonged RVVO gradually induces RV dilatation, leading to irreversible hemodynamic injury, which is not mitigated by right-side overload discharge.

Interestingly, EA patients with ASD had a significantly lower LVEDVI than healthy controls and EA patients without ASD. However, there was no significant difference in LVEDVI between healthy controls and EA patients without ASD. This might be attributed to the fact that the LV filling pressure of patients with EA generally remains within the normal range due to compensatory effects, and the LV filling pressure is impaired if the RVVO increases to a certain extent. Further, the EA with ASD group had a significantly lower LVEF and RVEF than the EA without ASD group. Although the patients without ASD had a lower LVEF than healthy controls, its average is still within the normal range (> 55%). We believe that RVVO coupled with RV pump dysfunction leads to a diminished RV output, thereby resulting in a reduced effective preload on the LV and a compromised LV cardiac output, which is consistent with the previous concept [21].

A previous study found that the presence of LV dysfunction could independently predict late mortality. Further, it was considered a high-risk factor for mortality after surgery [22, 23]. Therefore, the preservation, or reversal of LV dysfunction in EA could be a potential treatment target. Our previous research showed that LV PS derived via CMR tissue tracking could be an earlier and more comprehensive indicator of LV dysfunction than LVEF in EA [11]. In this study, the radial, circumferential, and longitudinal PS, and PDSR of patients with EA significantly decreased. In addition, the EA with ASD group had a significantly lower longitudinal PDSR than the EA without ASD and simple ASD groups. However, there was no significantly difference in terms of PSSR parameters among healthy controls, patients with ASD, and those with EA. Therefore, diastolic dysfunction is likely the predominate mechanism related to LV dysfunction in patients with EA, particularly in the subset of patients with ASD. This result is consistent with that of the study of Inai, which provided information about diastolic dysfunction in patients with EA who had preserved LVEF [24]. Thus, the normalization of loading conditions at end-diastole may be the main mechanism that can improve LV function.

### Limitations

The current study had several limitations. First, our cohort only comprised a small number of patients with EA with ASD, and the statistical power was limited. However, the number of patients with EA in this study was relatively larger than that in other published studies. Second, this was a retrospective study with all its inherent limitations. Not all patients with EA on echocardiography had data on the ASD size, which inhibited the further investigation of ASD effects on LV function. Finally, our patient groups might be biased because we studied only adult patients with EA and with ASD who were referred to CMR. As this was a cross-sectional study, larger studies with a longer observation period must be performed to evaluate the CMR-derived indices believed to be associated with specific outcomes.

## Conclusion

In conclusion, concomitant ASD is an important risk factor of LV dysfunction in patients with EA. Based on the LV strain parameters of patients with EA, mainly the PDSR decreased, indicating that the diastolic dysfunction is likely the predominate mechanism related to LV dysfunction. Hence, the normalization of loading conditions at end-diastole can mainly improve LV function in patients with EA.

### Supplementary Information

**Additional file 1: Table S1. **CMR Parameters between non-cyanotic EA + ASD Patients and cyanotic EA + ASD Patients.** Table S2.** Left Ventricle Strain Parameters Difference between non-cyanotic EA + ASD Patients and cyanotic EA + ASD Patients.

## Data Availability

The datasets generated and/or analyzed during the current study are available from the corresponding author on reasonable request.

## Abbreviations

EA: Ebstein’s anomaly
ASD: Atrial septal defect
CMR: Cardiovascular magnetic resonance
LV: Left ventricle
RV: Right ventricular
fRV: Right ventricle
TV: Tricuspid valve
TR: Tricuspid regurgitation
RVVO: Right ventricular volume overload
NYHA: New York Heart Association
LGE: Late gadolinium enhancement
EDVI: End-diastolic volume index
ESV: End-systolic volume
SV: Stroke volume
EF: Ejection fraction
PS: Peak strain
PSSR: Peak systolic strain rate
PDSR: Peak diastolic strain rate

## References

[1] Attenhofer Jost CH, Connolly HM, Dearani JA, Edwards WD, Danielson GK (2007). Ebstein’s anomaly. Circulation.

[2] Perdreau E, Tsang V, Hughes ML, Ibrahim M, Kataria S, Janagarajan K (2018). Change in biventricular function after cone reconstruction of Ebstein’s anomaly: an echocardiographic study. Eur Heart J Cardiovasc Imaging.

[3] Fratz S, Janello C, Müller D, Seligmann M, Meierhofer C, Schuster T (2013). The functional right ventricle and tricuspid regurgitation in Ebstein’s anomaly. Int J Cardiol.

[4] Fuchs MM, Connolly HM (2020). Ebstein anomaly in the adult patient. Cardiol Clin.

[5] Safi LM, Liberthson RR, Bhatt A (2016). Current management of Ebstein’s anomaly in the adult. Curr Treat Options Cardiovasc Med.

[6] Steinmetz M, Schuster A (2021). left ventricular pathology in Ebstein’s anomaly-myocardium in motion: cmr insights into left ventricular fibrosis, deformation, and exercise capacity. Circ Cardiovasc Imaging.

[7] Qureshi MY, Dearani JA (2021). Commentary: Gold or silver? Value of cardiac magnetic resonance imaging over echocardiography in Ebstein’s anomaly. J Thorac Cardiovasc Surg.

[8] Schulz-Menger J, Bluemke DA, Bremerich J (2020). Standardized image interpretation and post-processing in cardiovascular magnetic resonance—2020 update: Society for Cardiovascular Magnetic Resonance (SCMR): Board of Trustees Task Force on Standardized Post-Processing. J Cardiovasc Magn Reson.

[9] Bonow RO, Carabello BA, Chatterjee K, de Leon AC, Faxon DP, Freed MD (2008). Focused update incorporated into the ACC/AHA 2006 guidelines for the management of patients with valvular heart disease: a report of the American College of Cardiology/American Heart Association Task Force on Practice Guidelines (writing committee to revise the 1998 guidelines for the management of patients with valvular heart disease): endorsed by the Society of Cardiovascular Anesthesiologists, Society for Cardiovascular Angiography and Interventions, and Society of Thoracic Surgeons. Circulation.

[10] Kilner PJ, Geva T, Kaemmerer H, Trindade PT, Schwitter J, Webb GD (2010). Recommendations for cardiovascular magnetic resonance in adults with congenital heart disease from the respective working groups of the European Society of Cardiology. Eur Heart J.

[11] Liu X, Zhang Q, Yang ZG, Shi K, Xu HY, Xie LJ (2017). Assessment of left ventricular deformation in patients with Ebstein’s anomaly by cardiac magnetic resonance tissue tracking. Eur J Radiol.

[12] Tobler D, Yalonetsky S, Crean AM (2013). Right heart characteristics and exercise parameters in adults with Ebstein anomaly: new perspectives from cardiac magnetic resonance imaging studies. Int J Cardiol.

[13] Yang D, Li X, Sun JY (2018). Cardiovascular magnetic resonance evidence of myocardial fibrosis and its clinical significance in adolescent and adult patients with Ebstein’s anomaly. J Cardiovasc Magn Reson.

[14] Kheiwa A, Hari P, Madabhushi P, Varadarajan P (2020). Patent foramen ovale and atrial septal defect. Echocardiography.

[15] Arya P, Beroukhim R (2014). Ebstein anomaly: assessment, management, and timing of intervention. Curr Treat Options Cardiovasc Med.

[16] Stout KK, Daniels CJ, Aboulhosn JA, Bozkurt B, Broberg CS, Colman JM (2019). 2018 AHA/ACC guideline for the management of adults with congenital heart disease: a report of the American College of Cardiology/American Heart Association Task Force on Clinical Practice Guidelines. J Am Coll Cardiol.

[17] Nu S, Yang K, Chen X (2022). Cardiac remodeling after tricuspid valve repair in Ebstein’s anomaly: a magnetic resonance study. Eur Radiol.

[18] Badiu CC, Schreiber C, Hörer J, Ruzicka DJ, Wottke M, Cleuziou J (2010). Early timing of surgical intervention in patients with Ebstein’s anomaly predicts superior long-term outcome. Eur J Cardiothorac Surg.

[19] Goleski PJ, Sheehan FH, Chen SS, Kilner PJ, Gatzoulis MA (2014). The shape and function of the left ventricle in Ebstein’s anomaly. Int J Cardiol.

[20] Steinmetz M, Usenbenz S, Kowallick JT, Hosch O, Staab W, Lange T, Kutty S, Lotz J, Hasenfuss G, Paul T (2017). Left ventricular synchrony, torsion, and recoil mechanics in Ebstein’s anomaly: insights from cardiovascular magnetic resonance. J Cardiovasc Magn Reson.

[21] Kühn A, De Pasquale MG, Müller J, Petzuch K, Fratz S, Röhlig C (2013). Tricuspid valve surgery improves cardiac output and exercise performance in patients with Ebstein’s anomaly. Int J Cardiol.

[22] Brown ML, Dearani JA, Danielson GK, Cetta F, Connolly HM, Warnes CA (2008). Effect of operation for Ebstein anomaly on left ventricular function. Am J Cardiol.

[23] Chauvaud S, Berrebi A, d’Attellis N, Mousseaux E, Hernigou A, Carpentier A (2003). Ebstein’s anomaly: repair based on functional analysis. Eur J Cardiothorac Surg.

[24] Inai K, Nakanishi T, Mori Y, Tomimatsu H, Nakazawa M (2004). Left ventricular diastolic dysfunction in Ebstein’s anomaly. Am J Cardiol.

