# Supplementary material for: Effect of concomitant atrial septal defect on left ventricular function in adult patients with unrepaired Ebstein’s anomaly: a cardiovascular magnetic resonance imaging study
Source: J Cardiovasc Magn Reson. 2023 Dec 7;25:75. doi: 10.1186/s12968-023-00976-3 (PMC10702047; doi:10.1186/s12968-023-00976-3)
Supplement: Supplementary file 1 — Additional file 1: Table S1. CMR Parameters between non-cyanotic EA + ASD Patients and cyanotic EA + ASD Patients. Table S2. Left Ventricle Strain Parameters Difference between non-cyanotic EA + ASD Patients and cyanotic EA + ASD Patients. [file 12968_2023_976_MOESM1_ESM.doc]

**Additional material**

**Table S1. CMR Parameters between** **non-cyanotic EA + ASD Patients and cyanotic EA + ASD Patients**

|  | **non-cyanotic EA + ASD**  **n=18** | **cyanotic EA + ASD**  **n=13** | ***P* Value** |
| --- | --- | --- | --- |
| **LVEDVI, ml/m^2^** | 61.41(53.43-70.00) | 66.58(55.55-81.94) | 0.615 |
| **LVESVI, ml/m^2^** | 29.66(25.28-38.26) | 31.15(26.63-39.66) | 0.805 |
| **LVSVI, ml/m^2^** | 32.97±10.02 | 35.20±8.72 | 0.525 |
| **LVEF, %** | 49.09±8.23 | 50.86±7.23 | 0.539 |
| **RVEDVI, ml/m^2^** | 206.94±60.97 | 223.64±92.09 | 0.548 |
| **RVESVI, ml/m^2^** | 127.80(89.76-178.20) | 191.22(90.87-229.53) | 0.272 |
| **RVSVI, ml/m^2^** | 71.38±31.19 | 61.28±31.59 | 0.382 |
| **RVEF, %** | 34.99±12.30 | 28.61±10.89 | 0.148 |
| **RVEDV/LVEDV** | 3.38±1.33 | 3.34±1.47 | 0.944 |

Note: Data given as the mean ± SD or median (25th, 75th percentile). LV, left ventricular; RV, right ventricular; EDV, end diastolic volume; ESV, end systolic volume; SV, stroke volume; EF, ejection fraction; I, indexed to BSA.

**Table S2. Left Ventricle Strain Parameters Difference between non-cyanotic EA + ASD Patients and cyanotic EA + ASD Patients**

|  | **non-cyanotic EA + ASD**  **n=** | **cyanotic EA + ASD**  **n=** | ***P* Value** |
| --- | --- | --- | --- |
| **PS (%)** |  |  |  |
| Radial | 30.47±8.91 | 31.41±7.27 | 0.756 |
| Circumferential | -15.83±3.72 | -16.04±4.31 | 0.883 |
| Longitudinal | -9.47±4.13 | -7.91±7.04 | 0.446 |
| **PSSR (1/s)** |  |  |  |
| Radial | 1.81(1.61-2.13) | 1.89(1.34-2.05) | 0.363 |
| Circumferential | -0.91±0.25 | -0.95±0.22 | 0.656 |
| Longitudinal | -0.57(-0.76-(-0.43) | -0.58(-0.78-(-0.36) | 0.574 |
| **PDSR (1/s)** |  |  |  |
| Radial | -1.89(-2.59-(-1.60) | -1.89(-2.40-(-1.56)) | 0.780 |
| Circumferential | 1.17±0.43 | 1.09±0.37 | 0.571 |
| Longitudinal | 0.58(0.46-0.86) | 0.62(0.46-0.72) | 0.387 |

Note: Data given as the mean ± SD or median (25th, 75th percentile). PS, peak strain; PSSR, peak systolic strain rate; PDSR, peak diastolic strain rate.
